# Minimally Invasive Cardiac Surgery: A State-of-the-Art Review

**DOI:** 10.3390/jcm15010371

**Published:** 2026-01-04

**Authors:** Salvatore Poddi, Alessio Rungatscher

**Affiliations:** Division of Cardiac Surgery, University of Verona Medical Center, 37124 Verona, Italy; salvatore.poddi@univr.it

**Keywords:** minimally invasive cardiac surgery, mini-sternotomy, mini-thoracotomy, totally endoscopic surgery, robotic surgery, mitral valve, aortic valve, MIDCAB, TECAB, state-of-the-art review

## Abstract

Minimally Invasive Cardiac Surgery (MICS) was developed in the late 1990s and has taken giant strides over the last 30 years. Nowadays, it is an important part of the cardiac surgery practice, accounting for one-third of total heart surgeries globally, with remarkable results. It is mostly performed for Mitral Valve repair but also for Aortic Valve Replacement and coronary artery bypass. However, the chronological evolution that led MICS to its current status has never been properly described. The best format to share a historical journey is the state-of-the-art (SotA) review. To the best of our knowledge, there are no orthodox SotA reviews on MICS. The aim of our study is to describe the current key MICS techniques, their historical development, and to discuss their role in the future of our specialty.

## 1. Introduction

Minimally Invasive Cardiac Surgery (MICS) could be referred to as “any procedure not performed with full sternotomy and Cardio-Pulmonary Bypass (CPB) support” (by the Society of Thoracic Surgeons) or “a small chest wall incision that does not include the conventional full sternotomy” (by the American Heart Association) [1]. Chitwood defined it as a “philosophy” toward cardiac procedures, entailing specific strategies for different surgeries [2]. The final goal is to reduce invasiveness compared to the contemporarily considered standard technique (full-sternotomy access).

Over the last 30 years, cardiac surgery has evolved continuously, making an interesting technical journey. Today, MICS is an integral component of contemporary cardiac surgery, as it accounts for one-third of total heart surgeries globally [3]. Excellent results have been achieved, especially for Mitral Valve (MV) surgery [4,5]. The historical evolution that led us to the current status has never been accurately described. An accurate analysis of this journey would help us better understand the rationale behind these approaches and techniques, their suitability, advantages and disadvantages, how to improve them, and their potential role in the future. The best format to share this fascinating story is the state-of-the-art (SotA) review.

SotA reviews are not frequently used in medical education; sometimes, they are not labeled. Conversely, some authors used the label SotA improperly. We think that a SotA review should have its place in our field, as it is the best way to describe and discuss how a phenomenon evolves over time.

To the best of our knowledge, there are no proper SotA reviews on MICS. The aim of our study is to describe the current key MICS techniques (*this is where we are now*), their historical development (*this is how we got here*), and to discuss their role in the future of our specialty (*this is where we could go next*).

## 2. Methodology

We designed our study based on the 3-part argument that every SotA review should describe: *this is where we are now* (current status of the technique); *this is how we got here* (origin and evolution of the techniques); *this is where we could go next* (future directions).

To perform an accurate review, we decided to follow the 6-stage process for conducting a SotA review proposed by Barry et al. in 2022 [6,7,8]. The phenomenon to be addressed in the present SotA review is the MICS (Stage 1). The time frame referring to the current MICS knowledge and technique standards (*this is where we are now*) is January 2015 through October 2025, as after the mid-2010s, many centers started MICS programs, and publications increased (Stage 2). Primary research questions were: What was the gold standard for cardiac surgery? Was there any way to improve outcomes and/or decrease complications? Which ways have been followed? When did the new ways start being described? How have the new ways developed over the years? What is the future of these techniques? (Stage 3). Those questions refer to *how we got here* and *this is where we could go next*; to be addressed, our search strategy consisted of a systematic literature search through the PubMed database to identify relevant papers published between January 1995 and October 2025 (Stage 4). We decided to use the PubMed database as it is the most widely accessible and comprehensive source for biomedical literature, ensuring transparency and reproducibility of our approach. The following advanced search keywords and sentences were used: “minimally invasive cardiac surgery” [Title] OR “ministernotomy” [Title] OR “minithoracotomy” [Title] OR “transapical transcatheter aortic valve implantation” [Title] OR “totally endoscopic cardiac surgery” [Title] OR “robotic cardiac surgery” [Title]. Inclusion criteria included meta-analyses, review articles, descriptive cohort studies, and case reports referring to the topics identified through the previously listed keywords. Exclusion criteria were duplicates and studies clearly outside the scope of this review. Percutaneous procedures are excluded from our review as they are non-surgical procedures.

We initially found 1380 articles in total. As a first step of our search strategy, we read the titles of each study and selected the ones that specifically referred to our topics. As a second step, we read abstracts to select the potentially interesting papers. The latter were carefully read in full (as a third step) to include the most appropriate and impactful articles. To avoid bias, the search and selection process was carried out by two authors independently (SP and AR); after independent selections, only papers approved by both authors were included. An accurate analysis to discuss the relevance of every single article was then performed by the two authors. Articles’ references were reviewed to include additional relevant sources (Stage 5). Literature search, article selection, and analysis were performed between 1 September 2025 and 30 November 2025. At the end of the process, we decided to include 85 studies. A reflexive approach was adopted by the authors to ensure transparency and awareness of potential interpretive bias (Stage 6).

## 3. Minimally Invasive Cardiac Surgery: Current Status

MICS plays a major role in the current cardiac surgery practice. It is increasingly popular as the less invasive approach leads to better cosmetic results and, more importantly, better post-operative outcomes (less pain, shorter hospital stay, lower risk of skin infection, faster patient recovery) [4,5,9,10,11]. Among several MICS approaches and techniques, the most used today are mini-sternotomy (MS) and mini-thoracotomy approaches, the totally endoscopic (TE) technique, and the robotic (RT) technique.

**Figure 1:**
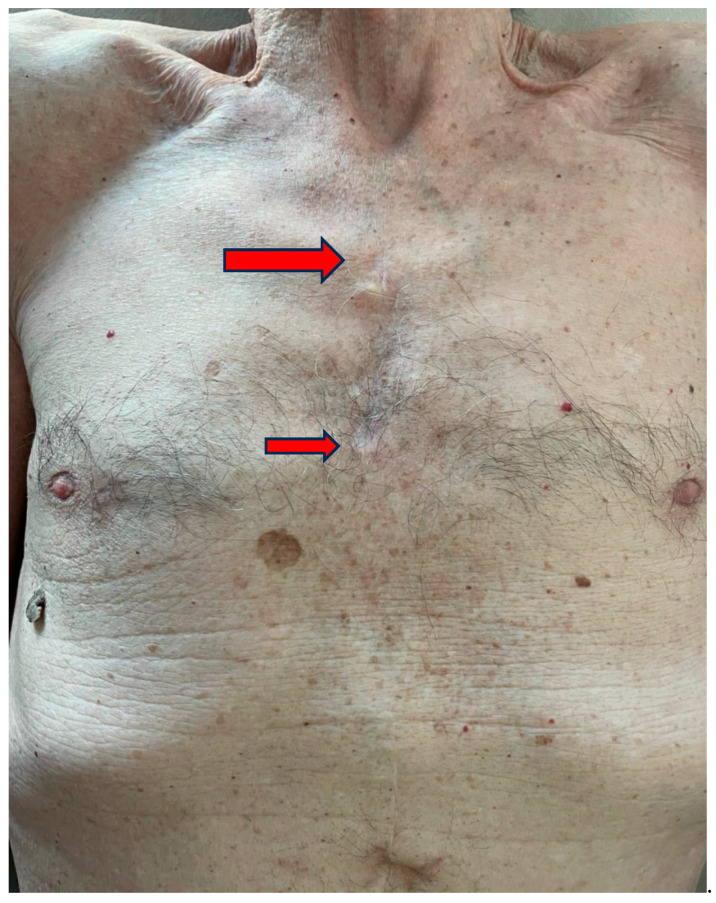
J-shaped, upper mini-sternotomy for Bentall procedure (Mini-Bentall). An incision was made from the angle of Louis (bigger arrow) through the third intercostal space (smaller arrow). Excellent cosmetic result.

MS (especially J-shaped upper mini-sternotomy; 5–6 cm incision; central or peripheral CPB cannulation) is nowadays well-known and widely diffused, mostly for Aortic Valve Replacement (AVR); outcomes are comparable to standard sternotomy and Right Mini-Thoracotomy (RMT), confirming the advantages of MICS [12,13,14]. MS has also been described for aortic root and arch surgery [15] (Figure 1).

RMT heart surgery (5–6 cm incision; peripheral CPB cannulation) can be performed both through direct-vision and video-assisted techniques; it is used for both AVR and MV surgery, once again demonstrating excellent results in terms of peri-operative and post-operative outcomes [16,17]. Some authors used this approach for isolated tricuspid valve (TV) surgery [18]. Lamelas et al. used this technique to perform combined AVR and Ascending Aorta Replacement (AAR), concluding that AVR+AAR were associated with longer operative times without increasing short-term morbidity or mortality compared with isolated AVR [17]. Left mini-thoracotomy, conversely, is used to perform both Trans-Apical Trans-Catheter Aortic Valve Implantation (TA TAVI) and the so-called Minimally Invasive Direct Coronary Artery Bypass grafting (MIDCAB). TA TAVI (hybrid, off-pump aortic valve implantation procedure) showed satisfactory short- to mid-term outcomes in patients deemed high-risk for standard surgery [19]. Maeda described satisfactory long-term outcomes as well [20]. MIDCAB (off-pump, single- or double-Coronary Artery Bypass Graft [CABG]) proved to be safe and effective and can be successfully performed in selected patients with low morbidity and excellent long-term results [21,22].

**Figure 2:**
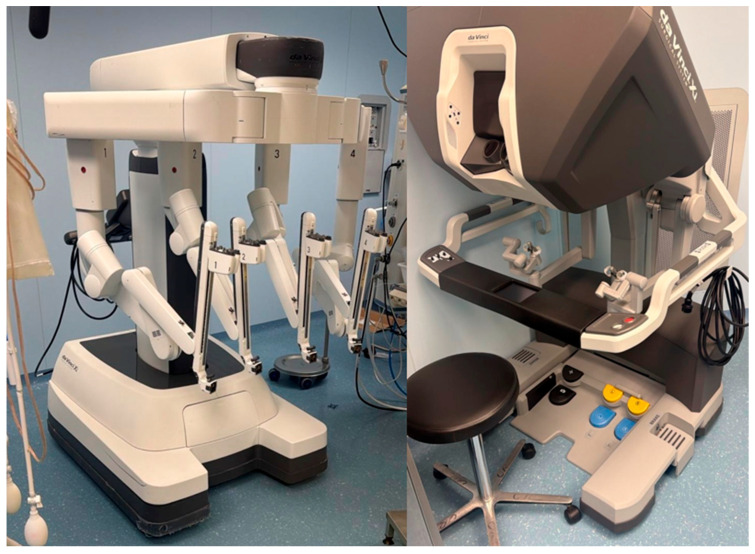
Intuitive Da Vinci Xi Surgical System. (**Left**): robot. (**Right**): console. Authors’ own photograph.

The two most fascinating and less invasive MICS techniques are TE and RT. TE cardiac surgery (totally video-guided heart surgery; main port: 3–4 cm incision; peripheral CPB cannulation) is increasingly adopted and has shown great outcomes, especially for MV surgery [9,23,24]. In some centers, it is now the standard of care for MV surgery [24]. Some authors used this technique to perform AVR [10,25,26]. Some centers gained experience with non-robotic, TE CABG [27]. Furthermore, there are case series and case reports about AAR and a concomitant triple valve and AAR surgery [26,28]. The RT platform (Figure 2) and technique (3-dimensional video-guided surgery; main port: 3–4 cm incision; peripheral CPB cannulation) showed excellent results as well, especially for MV surgery [4,5,11,29,30]. Gillinov and colleagues achieved a 99.5% MV repair (MVr) rate over their first 1000 robotic MV cases, with low mortality and morbidity; CPB and ischemic times shortened after the first 200 cases [4]. In their 10-year experience with robotic MV surgery, Mayo Clinic surgeons described excellent results, comparable with the standard technique [5]. Roach et al. claimed a 99% repair rate performing robotic MV surgery [29]. During RT, concomitant procedures such as TV surgery or Maze ablation can be performed [4,5,30]. Furthermore, some centers use RT for AVR and CABG surgery (TECAB), claiming great results [31,32,33,34].

Surgeons’ experience is a key factor in achieving excellent results, as the latter improve after the initial skill acquisition stage; patient selection is also crucial, as not all patients are suitable for MICS [4,35,36].

If we then look at the current status of MICS, we can state that it represents an important component of modern cardiac surgery, showing very good outcomes, particularly in MV surgery. The Mini-Mitral International Registry is the largest worldwide registry of minimally invasive MV surgery; its data confirmed excellent operative outcomes in low-, intermediate-, and high-risk patients, with very low mortality and morbidity rates [37].

## 4. Origin and Evolution of MICS

### 4.1. Mini-Sternotomy

MS was described as a less invasive approach for CABG in 1996 [38,39]. Arom published a 16-patient case series regarding single- or double-CABG, with or without CPB, through a 10–12 cm skin incision [39]. MS for AVR was described in 1997 by Szerafin: a 23-patient case series without intra- or post-operative complications; the authors concluded by recommending this technique for most AVRs [40]. In 1999, Byrne et al. described 137 aortic root replacement cases through “upper hemi-sternotomy,” concluding that this approach allows for a broad range of aortic surgeries with acceptable mortality and morbidity [41]. Later in the years, comparisons between full sternotomy and MS and a meta-analysis confirmed the good AVR results through MS [42,43,44].

### 4.2. Right Mini-Thoracotomy

RMT was first described by Carpentier in 1996. The French professor performed an MVr through a 5 × 4 cm skin access, with video assistance [45]. One year later, Chitwood published a 31-patient case series on MV surgery using this approach; early results suggested that surgery could be performed safely with low morbidity and earlier discharge [46]. In 2009, a triple-valve surgery was described [47]. RMT was also used for AVR, showing, in 2013, lower incidence of post-operative atrial fibrillation and blood transfusion, shorter ventilation time, and shorter hospital length of stay [48]. In 2013, Ward et al. published their 1922-patient experience with direct vision, RMT MVr, showing good results and stating that this was their standard of care approach for MV surgery [49]. A meta-analysis conducted by Sündermann in 2014 demonstrated that RMT and conventional MV surgery had similar peri-operative outcomes [50]. RMT was then described for other surgical scenarios, like MV endocarditis, concomitant AVR and CABG, and the Maze procedure [51,52,53].

### 4.3. Left Mini-Thoracotomy: TA TAVI and MIDCAB

TA TAVI was first described in 2006 as a less invasive alternative for patients at high surgical risk; after a left mini-thoracotomy, the prosthesis was inserted through an apical puncture and implanted within the native valve. Initial experience suggested its feasibility in selected patients with aortic valve stenosis [54]. In 2011, the PARTNER Trial showed that 1-year survival in high-risk patients was similar after surgical AVR or TAVI (both TA and transfemoral [TF]) [55]. MIDCAB through left mini-thoracotomy was described in 1995 by Benetti [56]. A few years later, in 1998, the CardioThoracic System registry of MIDCAB presented a 508-patient study that showed good early outcomes; however, long-term studies were needed [57]. In 2008, a meta-analysis by Kettering concluded that clinical outcomes were acceptable, but long-term data and trials were needed as well [58]. One year later, a comparison between single-CABG MIDCAB and off-pump CABG via sternotomy showed that MIDCAB could be performed safely, in selected patients, with low mortality [59]. In 2015, Reser published mid-term results, showing low mortality [60].

### 4.4. Totally Endoscopic Technique

The TE technique was the next logical step after direct-vision and video-assisted RMT, as the goal was to further minimize skin incision and improve field view. However, at the very beginning, that expression was uncommon. Probably the first reported TE was the “micro-mitral operation” by Chitwood in 1997, as he described a 2-inch skin incision (5 cm) and stated that the excision of the anterior leaflet, valve sutures, prosthesis seating, and knot tying were totally video-guided [61]. The Port-Access system (Cardiovations, Ethicon Inc., Somerville, NJ, USA) et similia included an endovascular aortic clamp, allowing a very small working port. In the subsequent years, some studies demonstrated its feasibility, while others showed higher procedural risks [62,63,64]. In 2003, the first TE AVR was described [65]. In 2005, comparisons between Port-Access and trans-thoracic clamp demonstrated that the latter shortened cross-clamp time, reduced peri-operative costs, and simplified the procedure [66,67]. In the 2010s, after developing specific trans-thoracic aortic clamps, the TE technique became more popular and showed all MICS advantages [68].

### 4.5. Robotic Technique

In 1998, two years after describing the first mini-thoracotomy MV surgery, Carpentier took one step forward, describing the first robotically assisted heart surgery on a 52-year-old woman with an aneurysm and a large defect of the interatrial septum [69]. One year later, Loulmet reported the first cases of robotic TECAB [70]. MV surgery quickly became the most practiced among robotic MICS. In 2000, the early Leipzig experience demonstrated that robotic MV surgery could be reliable and reproducible; they also described some concomitant Maze procedures [71]. The same year, both Grossi and Chitwood reported a robotic MVr case in the United States [72,73]. In 2004, Folliguet described the first case of robotic AVR [74]. In 2005, a United States multicenter trial (112 patients in 10 centers) showed that robotic MV surgery was safe and provided, as the greatest advantages, a magnified vision and tremor filtration; on the other hand, longer operative times were obvious [75]. To decrease CPB and cross-clamp times, the Cleveland Clinic presented a novel running-suture band annuloplasty technique in 2010, claiming shorter times [76]. In 2011, the Mayo Clinic published a comparative study evaluating standard versus robotic MV surgery. The authors concluded that the robotic approach enabled complete correction of all types of MV prolapse, using techniques that are fully equivalent to those used in conventional surgery. Moreover, robotic MVr showed excellent freedom from complications and earlier hospital discharge [77]. The same group, one year later, compared the quality of life (QoL) after standard vs. robotic MV surgery: the latter was associated with slightly improved early QoL and return to work [78]. Mayo Clinic, in 2013, also demonstrated that RT costs could be comparable with standard surgery [79]. In 2014, Yoo et al. showed mid-term mitral durability and improved surgical times over time [80]. The same year, the Emory group published early outcomes after robotic TECAB: it proved to be an effective alternative to standard CABG (even for multivessel disease) with comparable short-term outcomes [81].

## 5. Discussion and Future Directions

A SotA review seeks to create a critical summary of a current topic, describe its historical progression, and propose future directions [8]. To the best of our knowledge, this is the very first proper and orthodox MICS SotA review.

Full sternotomy had been the gold standard for heart surgery for over 50 years. Now something is changing. The concept of MICS arrived at the end of the 20th Century. There is no single, unanimous definition of MICS [1,2]. In our opinion, MICS should refer to every cardiac surgery approach and/or technique (so percutaneous procedures are excluded) that allows decreased physical trauma (smaller skin incisions, sternum and/or costal sparing), avoidance or reduction in invasive tools (CPB, cardioplegic arrest, hypothermia, mechanical ventilation, intubation, Intensive Care Unit [ICU] stay), shortened hospital stay, and prompt return to normal life. The final goal must be a “minimally invasive hospital experience” for the patient, without compromising short- and long-term outcomes.

**Figure 3:**
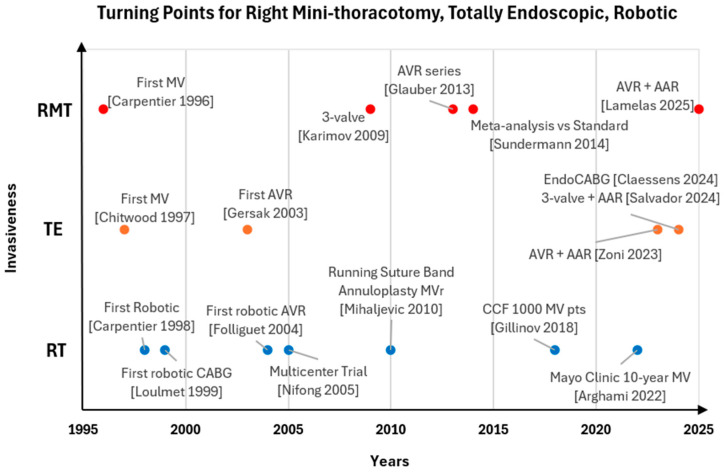
Turning points for Right Mini-Thoracotomy, Totally Endoscopic Technique, and Robotic Technique. In this figure, “invasiveness” refers to the approach and skin incision, as other invasive tools (e.g., cardiopulmonary bypass, aortic clamp) are used in all these techniques. AAR: Ascending Aorta Replacement. AVR: Aortic Valve Replacement. CABG: Coronary Artery Bypass Graft. CCF: Cleveland Clinic. MV: Mitral Valve. MVr: Mitral Valve repair. Pts: Patients. RMT: Right Mini-Thoracotomy. RT: Robotic Technique. TE: Totally Endoscopic Technique [4,5,17,26,27,28,45,47,48,50,61,65,69,70,74,75,76].

As previously described, several techniques have been developed over the past 30 years. Turning points of RMT, TE, and RT are depicted in Figure 3. Table 1 shows the main characteristics of those three approaches/techniques. MICS has become an integral part of modern cardiac surgery, and its relevance is anticipated to further increase in the coming years [2,3]. The most popular techniques are MS, RMT, TA TAVI and MIDCAB through left mini-thoracotomy, TE, and RT. Some approaches/techniques are procedure-specific (TA TAVI for aortic valve, MIDCAB for CABG), while others can be used for several surgeries. As a result, a broad spectrum of elective adult cardiac surgery is potentially covered by MICS, ranging from valve repair or replacement to CABG to aortic surgery.

MS demonstrated excellent results for AVR and aortic surgery, proving to be safe and showing shorter ICU and hospital stays, less pain, and decreased costs [12,13,14,15,43,44]. A great aspect of MS is that it is performed through standard surgical instruments, so no learning curve for specific tools is needed. CPB cannulation could be either central or peripheral based on anatomy and surgeon preference. For all these reasons, it can be stated that this approach should be the standard of care for elective AVRs and AARs; patient selection remains crucial, and, when appropriate, a surgeon should indicate a full sternotomy. The goal, in the future, should be to decrease as much as possible the full sternotomy approach among elective AVRs and AARs, restricting it to urgent/emergent or complex cases. On the other hand, MS spread could be affected by the increasingly popular TE and RT techniques.

RMT—both through direct vision and video-assisted- showed remarkable results mostly for MV surgery [16,17,49]. Its outcomes proved to be comparable with sternotomy, and some centers use RMT as a standard approach [49,50]. It may be adopted for most adult cardiac procedures, as it has also been applied to AVR, AAR, and CABG [17,52]. RMT has been an important benchmark over the last 20 years. However, it could become less popular in the near future. This approach has the same main advantages as TE (sternum sparing, magnified images if video-assisted), two common disadvantages (the learning curve for mastering long-shafted instruments in a limited environment, reduced tactile feedback), and some specific downsides, such as the bigger incision (potentially leading to more bleeding and infections) and no magnified images when the direct vision approach is chosen. For these reasons, RMT may give way soon, replaced by TE or RT for MV and probably AVR, and/or MS for AVR and AAR (depending on surgeon expertise and center experience).

TA TAVI was first performed in 2006 [54]. The PARTNER Trial showed similar 1-year survival between AVR and TAVI (both TA and TF) in high-risk patients [55]. Anyway, TA TAVI is more invasive than TF TAVI (as the latter is a non-surgical procedure) and less safe (more bleeding, higher mortality) [82]. The future role of TA TAVI could be as a second option surgery, sporadically indicated, mostly for patients who are high-risk for standard surgery and presenting contraindications to TF TAVI. Nevertheless, more sophisticated TF TAVI devices will be available in the future, further reducing TA TAVI indications. MIDCAB showed excellent long-term outcomes [21,22]. However, its future could be risky as well because of the continuous development of other MICS (TECAB) and Percutaneous Coronary Intervention (PCI). TECAB showed less bleeding and faster recovery than MIDCAB [83]. In a meta-analysis involving 7710 patients, MIDCAB showed similar mortality and morbidity and less target revascularization compared with PCI [84]. It can be anticipated that soon, outcomes of PCI and TECAB will further improve; in such a scenario, MIDCAB may eventually be superseded by these two techniques.

The TE technique can potentially replace the mini-thoracotomy approach, as it presents more advantages (smaller skin incision, magnified vision, less bleeding, less pain) and can cover the same broad surgery spectrum. Excellent results are described both for MV surgery and AVR [9,10,23,25,26]. Recently, endoscopic AAR and complex cases (triple-valve surgery) have been reported [26,28]. This technique will likely play a major role in the coming years, potentially becoming the standard of care for MV surgery and maybe rivaling MS for AVR and AAR. Robotic platforms are continuously evolving. Currently, the most popular robot is the DaVinci Surgical System (Intuitive Surgical, Sunnyvale, CA, USA). It has demonstrated superb results in MV surgery and, recently, in AVR and TECAB [4,5,29,30,31,32,33,34,83]. More recently, the world’s first fully robotic heart transplantation has been reported [85]. Compared to TE, RT has three major advantages: 3D, high-resolution, magnified view; tremor filtration; enhanced dexterity. These characteristics (confirmed by reported results) may permit RT to potentially cover all aspects of elective, adult cardiac surgery.

Technological innovations in cardiac surgery are progressing rapidly, and the long-term implications of emerging tools (like artificial intelligence) are difficult to predict. The development of artificial intelligence, however, could enable next-generation platforms to provide recommendations for the optimal technique in MVr or the appropriate prosthesis size in AVR. In the coming decades, robots could be capable of performing surgery autonomously, with surgeons present in the operating room only as a backup team. The potential role of robotic technology in the future of surgery is limited only by our imagination.

## 6. Limitations

This study has limitations. The aim of a SotA review is to give a chronological description of a specific topic. It may have lower reproducibility and exhaustiveness than a systematic review. The present review includes studies published after January 1995, searched through a single database. Our advanced search was designed to ensure a comprehensive and accurate review; however, some articles may have been missed due to keywords not included in the search strategy. Our review included case reports, whose statistical power is obviously lower than cohort studies and systematic reviews. Some studies refer to single-center data, limiting their external validity. Future, more robust studies are needed to compare long-term outcomes among the different techniques and improve diagnostic assessment, indication, and post-operative management.

## 7. Conclusions

MICS refers to all cardiac surgical approaches and techniques that reduce invasiveness compared with the traditional standard approach. Over the past three decades, several techniques have been developed. In recent years, TE and RT have been increasingly adopted, demonstrating excellent outcomes, particularly for MV surgery. In the coming years, these two techniques could be offered across a wide range of cardiac procedures, potentially becoming the standard of care for many of them.

## Figures and Tables

**Table 1:** Main features of Right Mini-Thoracotomy, Totally Endoscopic, Robotic.

	Right Mini-Thoracotomy	Totally Endoscopic	Robotic
Skin Incision	5–6 cm	3–4 cm	3–4 cm
Visualization	Direct vision Video-assisted	Video-guided	Video-guided
Surgeries	Usually MV also AVR, AAR, CABG	Usually MV also AVR, AAR, CABG	Usually MV also AVR, CABG
Technical Advantages	Sternal sparing Magnified vision when video-assisted	Sternal sparing Smaller incision than RMT Magnified vision	Sternal sparing Smaller incision than RMT 3D Magnified vision Tremor filtration Enhanced dexterity
Technical Disadvantages	Bigger incision than TE Learning curve for long-shafted instruments No magnified vision if direct vision	Learning curve for long-shafted instruments	Learning curve for robotic instruments

AAR: Ascending Aorta Replacement. AVR: Aortic Valve Replacement. CABG: Coronary Artery Bypass Graft. MV: Mitral Valve. RMT: Right Mini-Thoracotomy. TE: Totally Endoscopic.

## Data Availability

No new data were created or analyzed in this study. Data sharing is not applicable.

## Abbreviations

The following abbreviations are used in this manuscript:

AAR	Ascending Aorta Replacement
AVR	Aortic Valve Replacement
CABG	Coronary Artery Bypass Graft
CPB	Cardio-Pulmonary Bypass
ICU	Intensive Care Unit
MICS	Minimally Invasive Cardiac Surgery
MIDCAB	Minimally Invasive Direct Coronary Artery Bypass
MS	Mini-Sternotomy
MV	Mitral Valve
MVr	Mitral Valve Repair
PCI	Percutaneous Coronary Intervention
QoL	Quality of Life
RMT	Right Mini-Thoracotomy
SotA	State-of-the-Art
TA TAVI	Trans-Apical Trans-Catheter Aortic Valve Implantation
TE	Totally Endoscopic
TECAB	(robotic) Totally Endoscopic Coronary Artery Bypass
TF	Trans-Femoral (TAVI)
TV	Tricuspid Valve
RT	Robotic
